# A new long-read mitochondrial-genome protocol (PacBio HiFi) for haemosporidian parasites: a tool for population and biodiversity studies

**DOI:** 10.1186/s12936-024-04961-8

**Published:** 2024-05-04

**Authors:** M. Andreína Pacheco, Axl S. Cepeda, Erica A. Miller, Scott Beckerman, Mitchell Oswald, Evan London, Nohra E. Mateus-Pinilla, Ananias A. Escalante

**Affiliations:** 1 Biology Department/Institute of Genomics and Evolutionary Medicine (iGEM), Temple University, (SERC - 645), 1925 N. 12 St, Philadelphia, PA 19122-1801 USA; 2 University of Pennsylvania, Wildlife Futures Program, Kennett Square, Philadelphia, PA 19348 USA; 3 USDA Wildlife Services, Springfield, IL 62711 USA; 4 Department of Animal Sciences, University of Illinois at Urbana-Champaign, Champaign, IL 61801 USA; 5 Illinois Natural History Survey-Prairie Research Institute, University of Illinois at Urbana-Champaign, Champaign, IL 61820 USA; 6 Department of Natural Resources and Environmental Sciences, University of Illinois at Urbana-Champaign, Champaign, IL 61820 USA; 7 Department of Pathobiology, College of Veterinary Medicine, University of Illinois at Urbana-Champaign, Champaign, IL 61802 USA

**Keywords:** *Plasmodium*, *Haemoproteus*, *Leucocytozoon*, Machine learning, Mitochondrial genome, Mixed infection, Co-infections

## Abstract

**Background:**

Studies on haemosporidian diversity, including origin of human malaria parasites, malaria's zoonotic dynamic, and regional biodiversity patterns, have used target gene approaches. However, current methods have a trade-off between scalability and data quality. Here, a long-read Next-Generation Sequencing protocol using PacBio HiFi is presented. The data processing is supported by a pipeline that uses machine-learning for analysing the reads.

**Methods:**

A set of primers was designed to target approximately 6 kb, almost the entire length of the haemosporidian mitochondrial genome. Amplicons from different samples were multiplexed in an SMRTbell® library preparation. A pipeline (HmtG-PacBio Pipeline) to process the reads is also provided; it integrates multiple sequence alignments, a machine-learning algorithm that uses modified variational autoencoders, and a clustering method to identify the mitochondrial haplotypes/species in a sample. Although 192 specimens could be studied simultaneously, a pilot experiment with 15 specimens is presented, including in silico experiments where multiple data combinations were tested.

**Results:**

The primers amplified various haemosporidian parasite genomes and yielded high-quality mt genome sequences. This new protocol allowed the detection and characterization of mixed infections and co-infections in the samples. The machine-learning approach converged into reproducible haplotypes with a low error rate, averaging 0.2% per read (minimum of 0.03% and maximum of 0.46%). The minimum recommended coverage per haplotype is 30X based on the detected error rates. The pipeline facilitates inspecting the data, including a local blast against a file of provided mitochondrial sequences that the researcher can customize.

**Conclusions:**

This is not a diagnostic approach but a high-throughput method to study haemosporidian sequence assemblages and perform genotyping by targeting the mitochondrial genome. Accordingly, the methodology allowed for examining specimens with multiple infections and co-infections of different haemosporidian parasites. The pipeline enables data quality assessment and comparison of the haplotypes obtained to those from previous studies. Although a single locus approach, whole mitochondrial data provide high-quality information to characterize species pools of haemosporidian parasites.

**Supplementary Information:**

The online version contains supplementary material available at 10.1186/s12936-024-04961-8.

## Background

Haemosporidian parasite species (phylum Apicomplexa, order Haemosporida) are a diverse group of vector-borne protists that include the agents of malaria [1–4]. In addition to the species with known impact on human and veterinary health, these parasites infect various reptiles, mammals, and birds in almost all terrestrial ecosystems worldwide [2–5]. Although the Haemosporida are divided into four families (Plasmodiidae, Garniidae, Haemoproteidae, and Leucocytozoidae), most known species belong to three genera, *Plasmodium*, *Haemoproteus*, and *Leucocytozoon* [2–5].

Given their importance, there has been a renewed interest in species diversity, systematics, ecology, and distribution. Although a variety of loci have been used to study their evolutionary history and to perform the diagnostics of those species linked to human, wildlife, and veterinary diseases, most recent discoveries of new species have been driven using parasite mitochondrial genes, particularly the cytochrome b gene (*cytb*) [4, 6–13]. The wide use of this locus (*cytb* gene or mt genome) has been facilitated by its conservation across genera and its copy number, which favour its PCR amplification from various host species and samples of diverse qualities. Indeed, a 480 bp fragment of the *cytb* gene has become a de facto DNA barcode sequence for avian haemosporidians [4, 11, 13–15].

Despite the sensitivity and scalability of commonly used target gene approaches [16], there are still technical limitations wherever the parasite species pool is not defined, as is often the case in studies focusing on species discovery and diversity [4]. A common practice in biodiversity studies is to amplify a 480 bp *cytb* barcode fragment and perform direct Sanger sequencing, which often cannot separate different genetic lineages of the same parasite species (mixed infections) and/or species belonging to different species or genera (co-infections) that, indeed, are very common in wildlife [5, 17–26]. These mixed and/or co-infections may lead to chimeras or “consensus” sequences when the PCR amplicon is sequenced directly, which do not represent a reproducible lineage.

In addition, the small *cytb* gene fragment has limited informative sites, affecting phylogenetic inferences [13], a necessary step for describing newly discovered species. Due to direct sequencing specimens with mixed or co-infections, ambiguities are usually handled as gaps, Ns, or IUPAC codes, limiting the number of informative sites even further. This DNA barcoding approach provides valuable insights into haemosporidian diversity (revised in [4]), still, lineages with such limited information are used as a proxy for species when studying biodiversity patterns.

As an alternative, the complete linear mitochondrial genome (mt) with approximately 6 kb or partial sequences, including the three coding mt genes *cox1*, *cox3*, and *cytb*, have been used [4]. The mt genome is not saturated, yielding well-supported phylogenies [4, 12]. In addition, mitochondrial genes have comparable codon usages and AT content across taxa [12]; thus, there is a low risk of model misspecification when used in phylogenetic analyses [4, 12]. Finally, it does not recombine and allows population-level analyses to understand species’ evolutionary history and dynamics, as demonstrated in human and non-human primate parasites [27–29]. However, generating such data from large numbers of samples is costly and labor-intensive, particularly if mixed infections and/or co-infections require cloning. Although cloning yields accurate haplotypes, it may miss variants in low frequency simply because it is costly to sequence multiple clones per sample. Although short-read sequencing technologies are ideal for identifying single nucleotide polymorphisms, they may not solve the issue of reconstructing haplotypes in a sample with an unknown number of lineages such as those with mixed infections and/or co-infections [13].

Advances in Next-Generation Sequencing technology using long-reads have opened possibilities for target sequencing. One of the most recent technologies, the PacBio® HiFi sequencing method, generates long-read sequencing datasets (10–25 kb) with accuracies around 99.5%, making it an alternative to next-generation short reads sequencing technologies and Sanger sequencing, particularly with adequate coverage. This technology allowed for improvement in the quality of the results for genome assembly (metagenomes [30] and mitogenomes [31]) and the identification of single nucleotide polymorphism and structural variant detection.

Here, a PacBio HiFi protocol and a pipeline based on a machine learning method (HmtG-PacBio Pipeline) are developed for the amplification and read processing of mitochondrial genomes (≤ 6 kb) belonging to different genera of haemosporidian parasites. This method allows for accurate detection of mixed and/or parasite co-infections, including parasite lineages/haplotypes present in very low parasitaemia in different vertebrate hosts, which the standard *cytb* gene protocol cannot detect.

## Methods

### Design of barcoded oligonucleotides

Oligonucleotides (oligos) were designed using those previously reported as forward AE170 and reverse AE171 [12, 13]. These oligos have been successfully used to amplify the mt genomes (≤ 6 kb) of many species belonging to several haemosporidian genera from different vertebrate hosts (Table 1, [12, 13, 25, 26, 32–44]). Thirty-two barcoded oligos (Table 2), eight forwards, and 24 reverses were designed and tested. Each oligo contained a 5′ buffer sequence (GCATC), a 16-base barcode, and the slightly modified external oligos forward AE170 (5′-GAT TCT CTC CAC ACT TCA ATT CGT ACT TC`-3′) or reverse AE171 (5′-GAA AAT WAT AGA CCG AAC CTT GGA CTC-3′) sequences. All oligos contained 5′ phosphates and were obtained using HPLC-purification. Oligos were resuspended in nuclease-free water for molecular biology research (Sigma-Aldrich^®^ Solutions, Darmstadt, Germany) and stored at high concentration (100 μM) at −20 °C, avoiding repeated freeze thaws. The combination of these sets of primers can be used in all possible asymmetric pairs for multiplexing up to 192 different samples (eight forwards combined with 24 reverses).

**Table 1:** Number of parasites species belonging to different haemosporidian genera and vertebrate host that have been amplified using primers mt_AE170F and mt_AE171R

Vertebrate host	Minimum No. of species	Genus	References
Class: Mammalia			
Humans	4	*Plasmodium*	[25, 32, 33]
Apes	5	*Plasmodium*	
Macaques	12	*Plasmodium*	[12, 26]
Macaques	1	*Hepatocystis*	[12, 26]
Orangutan	2	*Plasmodium*	[12, 26]
Mandrills	2	*Plasmodium*	[12, 26]
Lemurs	10	*Plasmodium*	[34, 35]
Rodents	2	*Plasmodium*	[34]
Ruminant	1	*Plasmodium*	[35]
Class: Aves			
Birds	32	*Plasmodium*	[12]
	24	*Haemoproteus* (*Parahaemoproteus*)	[12]
	4	*Haemoproteus* (*Haemoproteus*)	[12, 36]
	1	*Haemoproteus catharti*	[37]
	1	*Haemoproteus pulcher*	
	12	*Leucocytozoon*	[37–41]
Class:Reptilia			
Turtle	1	*Haemocystidium*	[42]
Lizards	2	*Haemocystidium*	
	9	*Plasmodium*	[12, 43, 44]
Total	125

**Table 2:** Barcoded sequences of the target-specific forward (F) and reverse (R) primers for the amplification of the Haemosporida mt genome (≤ 6 kb)

Name	Sequence
AE170PB1_F	GCATCCACTCGACTCTCGCGTGATTCTCTCCACACTTCAATTCGTACTTC
AE170PB2_F	GCATCTCTGTATCTCTATGTGGATTCTCTCCACACTTCAATTCGTACTTC
AE170PB3_F	GCATCACAGTCGAGCGCTGCGGATTCTCTCCACACTTCAATTCGTACTTC
AE170PB4_F	GCATCACACTAGATCGCGTGTGATTCTCTCCACACTTCAATTCGTACTTC
AE170PB5_F	GCATCCGCATGACACGTGTGTGATTCTCTCCACACTTCAATTCGTACTTC
AE170PB6_F	GCATCCACGACACGACGATGTGATTCTCTCCACACTTCAATTCGTACTTC
AE170PB7_F	GCATCCACTCACGTGTGATATGATTCTCTCCACACTTCAATTCGTACTTC
AE170PB8_F	GCATCCATGTAGAGCAGAGAGGATTCTCTCCACACTTCAATTCGTACTTC
AE171PB1_R	GCATCAGAGACTGCGACGAGAGAAAATWATAGACCGAACCTTGGACTC
AE171PB2_R	GCATCCAGAGAGTGCGCGCGCGAAAATWATAGACCGAACCTTGGACTC
AE171PB3_R	GCATCCGCGCGTCGTCTCAGCGAAAATWATAGACCGAACCTTGGACTC
AE171PB4_R	GCATCAGAGAGTACGATATGTGAAAATWATAGACCGAACCTTGGACTC
AE171PB5_R	GCATCTCTGTAGTGCGTGCGCGAAAATWATAGACCGAACCTTGGACTC
AE171PB6_R	GCATCATGTGCGTGTGTGTCTGAAAATWATAGACCGAACCTTGGACTC
AE171PB7_R	GCATCCTCTCAGACGCTCGTCGAAAATWATAGACCGAACCTTGGACTC
AE171PB8_R	GCATCTATCTCAGTGCGTGTGGAAAATWATAGACCGAACCTTGGACTC
AE171PB9_R	GCATCTGTGTCTATACTCATCGAAAATWATAGACCGAACCTTGGACTC
AE171PB10_R	GCATCTATAGACTATCTGAGAGAAAATWATAGACCGAACCTTGGACTC
AE171PB11_R	GCATCGTATGTGAGAGAGCGCGAAAATWATAGACCGAACCTTGGACTC
AE171PB12_R	GCATCCACGCGACGCTCTCTAGAAAATWATAGACCGAACCTTGGACTC
AE171PB13_R	GCATCGAGAGCGCGAGTGCACGAAAATWATAGACCGAACCTTGGACTC
AE171PB14_R	GCATCGTGCTCTGTGTGTCACGAAAATWATAGACCGAACCTTGGACTC
AE171PB15_R	GCATCTGCGTGTATGTCATATGAAAATWATAGACCGAACCTTGGACTC
AE171PB16_R	GCATCACGAGATACTCGCGCGGAAAATWATAGACCGAACCTTGGACTC
AE171PB17_R	GCATCCTGTGTAGAGAGCACAGAAAATWATAGACCGAACCTTGGACTC
AE171PB18_R	GCATCTGATGTGACACTGCGCGAAAATWATAGACCGAACCTTGGACTC
AE171PB19_R	GCATCACTACTGAGACATAGAGAAAATWATAGACCGAACCTTGGACTC
AE171PB20_R	GCATCTATATCGCGTCGCTATGAAAATWATAGACCGAACCTTGGACTC
AE171PB21_R	GCATCGCGTACTGCGACTGTGGAAAATWATAGACCGAACCTTGGACTC
AE171PB22_R	GCATCATATATGCACGCTCTAGAAAATWATAGACCGAACCTTGGACTC
AE171PB23_R	GCATCCGCTGTATACACGCTCGAAAATWATAGACCGAACCTTGGACTC
AE171PB24_R	GCATCAGAGACTGTAGCGCACGAAAATWATAGACCGAACCTTGGACTC

The combination of these set of primers can be used in all possible asymmetric pairs for multiplexing up to 192 different samples. Oligos must contain 5′ phosphates, and HPLC-purification is highly recommended

### Samples, DNA extraction, parasite mt genome amplification, and library preparation

Fifteen positive archived blood samples from different vertebrate hosts (mammals, birds, and reptiles) were selected to test the oligos and this PacBio protocol (Table 3). Specifically, raptor and reptile samples were recently collected by Dr. Erica A. Miller (Wildlife Futures Program, University of Pennsylvania), USDA Wildlife Services, and Dr. Aaron Bauer (Villanova University), respectively, as part of an ongoing collaboration. Each sample was previously screened for haemosporidian parasites by microscopy [2, 3] and/or polymerase chain reaction (PCR), using primers targeting the complete *cytb* gene, which have been used in previous studies [13, 26, 34, 45, 46]. These samples were positive for haemosporidian parasites belonging to three genera (*Leucocytozoon*, *Haemoproteus*, and *Plasmodium*), and several of them already have the mt genome sequences available in GenBank, so they were considered ideal for testing the method [12, 26, 32]. Genomic DNA was extracted from whole blood using the DNeasy Blood & Tissue Kit (Qiagen, GmbH, Hilden, Germany), and the mt genome amplification was carried out with the TaKaRa LA Taq Polymerase (TaKaRa Mirus Bio Inc.) following manufacturers’ directions [12].

**Table 3:** Vertebrate host samples infected with different Haemosporida genera used to test the PacBio HiFi protocol

Vertebrate host (Sample)	Sanger sequencing (Strain or lineage)	PacBio sequencing	GenBank accession number
1-*Homo sapiens* (mammal)	*P. falciparum* (CDC: Ghana 3)	*P. falciparum* (single infection)	PP317143
2-*Homo sapiens* (mammal)	*P. vivax* (CDC: Sumatra)	*P. vivax* (single infection)	PP317144
3-*Homo sapiens* (mammal)	*P. vivax* (CDC: Mauritania I)	*P. vivax* (single infection)	PP317145
4-*Homo sapiens* (mammal)	*P. ovale* (CDC)	*P. ovale* (single infection)	PP317149
5-*Homo sapiens* (mammal)	*P. malariae* (CDC)	*P. malariae* (single infection)	PP317148
6-*Macaca* sp. (mammal)	*P. cynomolgi* (CDC: Mulligan)	*P. cynomolgi* (single infection)	PP317146
7-*Macaca* sp. (mammal)	*P. inui* (CDC: Taiwan II)	*P. inui* (single infection)	PP317147
8-*Agama aculeata* (reptile)	*Plasmodium* sp. (AMB10683)	*Plasmodium* sp. (single infection)	PP317150
9-*Pandion haliaetus* (bird)	*Plasmodium* sp. (MYCAME02)	***Plasmodium*** **sp. (MYCAME02)/*P. elongatum*** **(GRW06)**	PP317153/PP317154
10-*Megascops asio* (bird)	*Plasmodium* sp. (PADOM11)	*Plasmodium* sp. (PADOM11, single infection)	PP317151
11-*Cathartes aura* (bird)	*Haemoproteus catharti* (CATAUR01)	*Haemoproteus catharti* (single infection)	PP317156
12-*Bubo virginianus* (bird)	*Haemoproteus* sp. (STVAR01)	*Haemoproteus* sp. (STVAR01, single infection)	PP317157
13-*Buteo jamaicensis* (bird)	*P. elongatum* (PADOM11)	***P. elongatum*** ***(PADOM11)/****Leucocytozoon* **sp. (BUTJAM19)**	PP317152/PP317158 /PP317159
14-*Buteo jamaicensis* (bird)	*Leucocytozoon* sp. (BUTREG01)	***Leucocytozoon*** **sp. (BUTREG01)/*Leucocytozoon*** **sp. (BUTJAM19)**	PP317166/PP317160
15-*Buteo jamaicensis* (bird)	*Plasmodium* sp. (BT7)	***Plasmodium*** **sp. (BT7)/*Leucocytozoon*** **sp. (BUTJAM19 and BUTJAM20)**	PP317155/PP317161/ PP317162/PP317163/ PP317164/PP317165

Results of the traditional Sanger sequencing/cloning and PacBio HiFi sequencing are shown for comparation. GenBank accession number for mt genome sequences obtained in this study are also given. Mixed or coinfection are shown in bold

Three independent PCRs were performed for each sample using 3 µl of DNA and a unique oligo combination for each sample (Table 2). All PCR reactions were carried out in 50 μl volumes, and negative controls (dH_2_O) and positive controls (samples from an infected human) were included. Amplification conditions for all PCRs were: a partial denaturation at 94 °C for 1 min and 30 cycles with 30 s at 94 °C and 7 min at 68 °C, followed by a final extension of 10 min at 72 °C. PCR products were visualized in 1% LE analytical grade agarose (Promega Corporation, USA) gels and stained with GelRed® Nucleic Acid Gel Stain (Biotiun, San Francisco-California, USA). All three independent PCR products (50 μl) were excised from the gel (bands of ∼6 kb) and purified using the QIAquick Gel extraction kit (Qiagen, GmbH, Hilden, Germany). This last step is optional but highly recommended to get a cleaner PCR product for the library preparation.

**Fig. 1:**
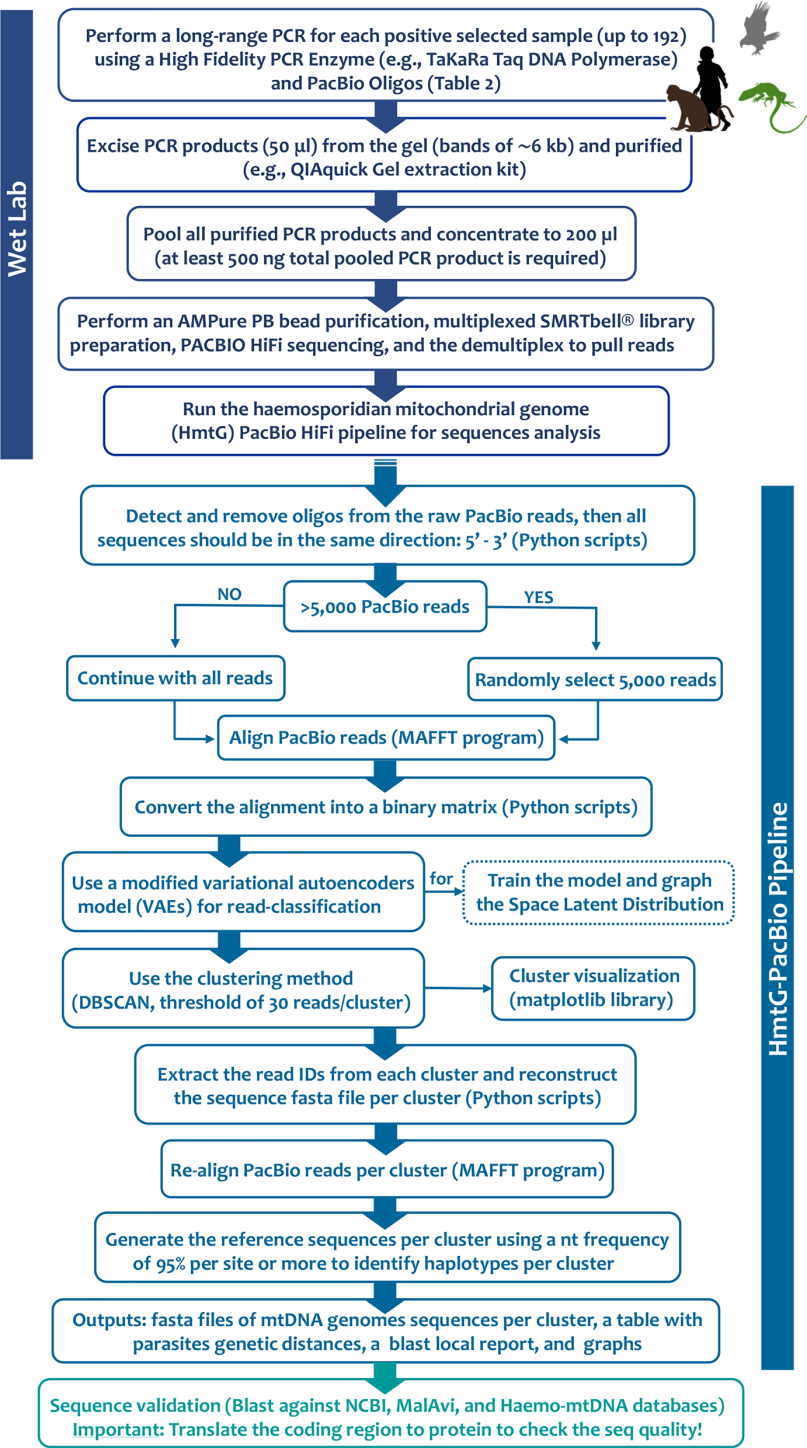
Flowchart of the Haemosporidian mt genome PacBio HiFi sequencing protocol and HmtG-PacBio Pipeline

Given that the parasitaemia varied between samples, all purified PCR products (50 µl x replicate x samples) were pooled in a clean 2.0 ml DNA LoBind microcentrifuge tube to ensure enough product for each sample (Eppendorf, Hamburg, Germany). Then, this pool was concentrated to 200 µl. The total DNA concentration was measured using a Qubit 3.0 fluorometer (Thermo Fisher Scientific, Massachusetts, USA), with a total amount of DNA of the pool being 5720 ng (28.6 ng/µl). Notice that at least 500 ng pooled PCR product is required for SMRTbell library preparation. Then, the pool was dried and sent to the DNA Services of the University of Illinois at Urbana-Champaign, Roy J. Carver Biotechnology Center (Urbana, IL 61801) for the AMPure PB bead purification, multiplexed SMRTbell® library preparation, sequencing, and the demultiplex to pull reads from the samples included in this study (Fig. 1). In brief, amplicons were converted to a library with the SMRTBell Express Template Prep kit 3.0. Then, the library was sequenced on 1 SMRTcell 8 M on a PacBio Sequel IIe using the CCS sequencing mode and a 30hs movie time. CCS analysis was done using SMRTLink V11.0 with the following parameters: ccs -min-passes 3-min-rq 0.999 and lima -ccs-preset HIFI-ASYMMETRIC-split-bam-named.

### Haemosporidian mitochondrial genome PacBio HiFi pipeline (HmtG-PacBio pipeline)

A pipeline was developed to analyze the mt genome sequences obtained by PacBio HiFi sequencing incorporating a machine-learning method. In particular, the pipeline integrates (1) custom Python scripts, (2) the multiple sequence alignment program MAFFT [47], (3) a modified variational autoencoders (VAEs) [48], and (4) a clustering method using DBSCAN algorithm (Density-Based Spatial Clustering of Applications with Noise) for data analysis and pattern recognition [49]. The pipeline, including all described scripts, is available on GitHub at https://github.com/EscalanteLab/HmtG-PacBio-Pipeline.git (Fig. 1).

**Fig. 2:**
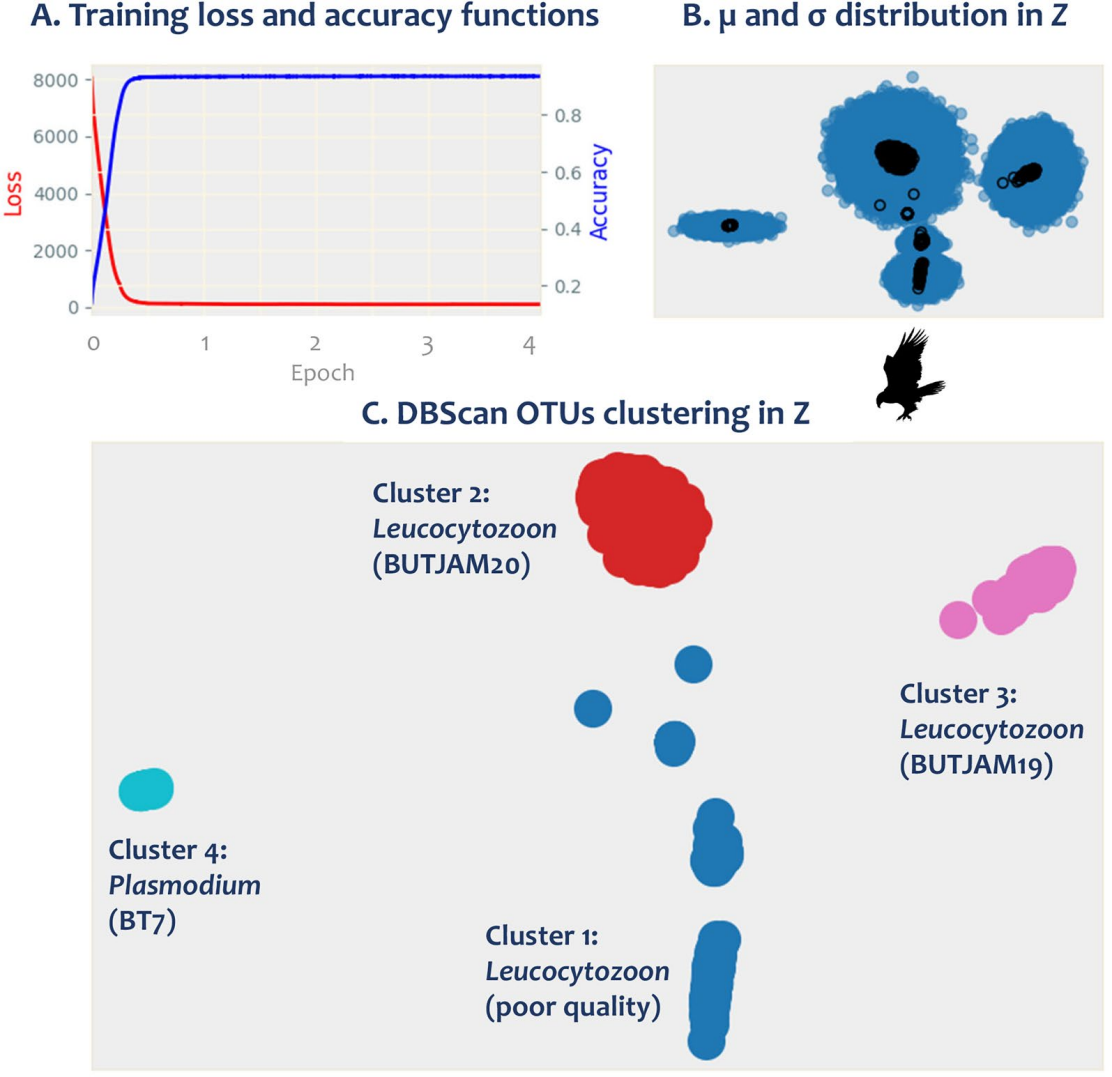
HmtG-PacBio Pipeline graph output. **A** Visualization of the training process, **B** mean (μ: black unfilled dots) and standard deviation (σ: blue filled dots) in *Z*, and **C** DBScan OTUs clustering in *Z* of the sample 15 which belong to a red-tailed hawk (*Buteo jamaicensis*, Accipitridae, Accipitriformes). See Table 3 for details

The variational autoencoders (VAEs) are a generative machine-learning model that discovers hidden patterns, such as putative groups of haemosporidian mt lineages/species [48]. The input data for the VAEs is an alignment converted into a binary matrix. In particular, after the oligos are detected/removed and all sequences are in the same orientation (5′ to 3′, Fig. 1) as implemented in this pipeline, nucleotides are transformed into unique four-dimensional binary variables (e.g., A = 1,0,0,0; C = 0,1,0,0; G = 0,0,1,0; T = 0,0,0,1) with gaps also included (e.g., “−” = 0,0,0,0) through a custom script. Within the VAEs, the encoder infers a distribution of latent variables (in this case, putative haplotypes) from the encoded variant sites as a normal distribution with a mean (μ) and a standard deviation (σ). Then, the decoder uses this inferred latent space (or Z) distribution to reconstruct the original encoded variant sites. As a result, distributions of reads/sequences are estimated in which reads resembling each other are positioned closer to one another in this lower dimension latent space (or Z). To evaluate the performance of the VAEs model, training functions such as loss (difference between the predicted value by the model and the true value, Fig. 2A) and accuracy (method for measuring a classification model’s performance, Fig. 2B) were used. This method was implemented using the Keras deep learning library in Python (https://keras.io; [50]) and the TensorFlow machine-learning framework (www.tensorflow.org; [51]).

Then, the DBSCAN algorithm is used to cluster that data, reducing the reads into groups or clusters with parameters set to eps = 1 (this parameter specifies how close points should be to each other to be considered a part of a cluster) and min_samples = 30 [49]; thus, only clusters with 30 reads or more are considered. Since the goal is to get clusters for each hypothetical haplotype or potential species included in the data, eps values less than 1 or greater than 2 are not recommended for this protocol. The 30-sequence cutoff was selected to ensure the reference sequences achieve a minimum of 30X coverage, guaranteeing reliable haplotype calling using long-read sequences [52]. Although the eps and min_samples parameters can be adjusted if needed, the parameters used here allow for the separation and clustering of long-read sequences at the species level (see Results section, Fig. 2).

This pipeline generates a minimum of 4 output files if the sample harbors a single infection without undetectable low-frequency variants (see below). One file contains the visualization of the training process (Fig. 2A), the μ and σ distribution in *Z* (Fig. 2B), and the DBScan OTUs clustering in *Z* (Fig. 2C). Graphs are obtained by using the matplotlib library in Python [53]. Figure 2B shows the latent space (*Z*) distribution of encoded sequences defined by their mean (μ: black unfilled dots) and standard deviation (σ: blue filled dots). For each cluster, a reference sequence is constructed using a 95% nucleotide frequency threshold per site. If a site does not exceed this threshold, it is masked. The correction process involves comparing each read from the cluster against this reference sequence. In cases where a site is masked, the nucleotide from the original read is preserved. Following this correction, the number of haplotypes is quantified considering a minimum 30 × threshold (the user can modify it if needed) for enhanced statistical confidence in genomic analysis [54, 55]. This level of coverage ensures greater accuracy in identifying genomic variation rather than random sequencing errors. Haplotypes below this threshold are excluded from primary analysis but retained as low-frequency variants in a separate text file for each cluster found in each sample.

The second file contains fasta outputs with all aligned haplotype sequences belonging to each cluster (one file for a sample with a single infection and more than one belonging to each cluster detected in a sample with mixed or coinfection). Then, two additional files are generated. One contains the pairwise genetic distances (p-distances) within and between clusters (if multiple clusters are presented in the sample), and the other includes the results from a local blast analysis of the final sequences obtained from each cluster found in each sample. This analysis utilizes a set of mitochondrial genome sequences available on GitHub at https://github.com/EscalanteLab/HmtG-PacBio-Pipeline.git, which is a compilation and extensive curation of the published mt genome sequences already deposited in GenBank [56]. Although this basic mt genome set will be regularly updated, users can update it independently if they have unpublished data or more recent data that they need to consider. The genetic distance and Blast results may provide the user with information that may facilitate genus/species identification. Still, they are not intended as a criterion for species delimitation or identification.

An extra file with the low-frequency haplotypes could be provided if they are present in the sample (less than 30 reads). Keeping the unconfirmed low-frequency variants allows the user to determine whether they could be actual infections in low frequency rather than sequencing errors. For example, the same haplotype could be present in more than one individual, or the variant could be a synonymous substitution, suggesting an actual infection since they are unlikely events assuming that sequencing errors are expected to be random or concentrated in tandem repeats in low complexity regions.

### Sequence analysis

To validate the quality of the sequences, a phylogenetic analysis was performed using Bayesian and Maximum Likelihood methods after the sequences were compared against the sequences available in GenBank [56] and MalAvi [11] databases using BLAST. For this analysis, the closest mt genome sequences to the sequences obtained in this study found by BLAST (identities > 99%) were downloaded and aligned with all sequences obtained here using ClustalX v2.0.12 and Muscle as implemented in SeaView v4.3.5 [57] with manual editing. This alignment (5388 bp excluding gaps) included 43 partial mt genome sequences belonging to three genera (*Leucocytozoon*, *Haemoproteus*, and *Plasmodium*).

Then, the phylogenetic relationships were inferred on this alignment using a Bayesian method implemented in MrBayes v3.2.7 with the default priors [58] and the maximum likelihood method implemented in IQ-TREE v2.3.1 [59]. A general time-reversible model with gamma-distributed substitution rates and a proportion of invariant sites (GTR + Γ + I) was used for the Bayesian method. This model had the lowest Bayesian Information Criterion (BIC) scores for this alignment, estimated using MEGA v7.0.14 [60]. Posterior probabilities for the nodes were inferred by sampling every 500 generations from two independent chains of 3 × 10^6^ Markov Chain Monte Carlo (MCMC) steps. Chains were assumed to have converged once the value of the potential scale reduction factor (PSRF) was between 1.00 and 1.02, and the average standard deviation of the posterior probability was < 0.01 [58]. Then, a “burn-in” of 25% of the sample was discarded. In the ML analysis, GTR + F + I + G4 was the substitution model obtained with ModelFinder [61] as implemented in IQ-TREE [59]. Support values were generated through Ultrafast bootstrap approximation (UFBoot) [62] with 1000 replicates. Both phylogenetic trees were compared and visualized using FigTree v1.4.4 (http://tree.bio.ed.ac.uk/software/figtree/).

### Statistical analysis of genetic distance results and putative sequencing/PCR errors

The expected PacBio HiFi sequencing error was first estimated using the Phred read quality scale or Q-value, which correlates with the probability of an error in base identification for each read. The accuracies are reported as Q value =  −0 × log10(P), where P is the measured error rate. Second, the observed sequencing error was calculated as a percentage of changes by comparing each raw read against the final sequence generated for each cluster divided by the total read length.

### In silico experiments to test the accuracy of the HmtG-PacBio pipeline

Given that there is a high prevalence of mixed haemosporidian infections and/or coinfections in wild vertebrate host populations [5, 17–26], two in-silico experiments were designed to explore the accuracy of this pipeline in separating lineages/haplotypes or species belonging to each resulting cluster from samples with mixed infections or/and co-infections. First, all obtained reads from six different samples, each one corresponding to a different well-known *Plasmodium* species infecting primates, including humans (specifically, 1-*Plasmodium falciparum*, 2/3-*Plasmodium vivax*, 4-*Plasmodium ovale*, 5-*Plasmodium malariae*, 6-*Plasmodium cynomolgi,* and 7-*Plasmodium inui;* Table 3), were pooled and the pipeline was run on this aggregated sample. Second, all reads belonging to four raptor samples (9-*Pandion haliaetus,* 11-*Cathartes aura*, 12-*Bubo virginianus*, and 14-*Buteo jamaicensis;* Table 3) were also pooled and reanalysed. Then, the clusters/sequences obtained from both in-silico experiments were compared to the original clusters/sequences obtained from each independent sample.

## Results

### Accuracy of the HmtG-PacBio pipeline

Clusters with more than 30 reads with an accuracy of 99.7% were obtained for all the selected samples (between 135 and 16742 reads, depending on the parasitaemia of the sample). Sample 15 belonging to a Red-tailed Hawk (*Buteo jamaicensis*, Accipitridae, Accipitriformes, Table 3) was selected to show PacBio HiFi results using HmtG-PacBio Pipeline. Figure 2 shows the visualization of the training process (Fig. 2A), the μ and σ distribution in *Z* (Fig. 2B), and DBScan OTUs clustering in *Z* (Fig. 2C) graphs for this sample.

The loss function that assesses the Variational Autoencoders (VAEs) model’s reconstruction capability showed a significant drop after the first epoch (one complete cycle of the training dataset through the VAE model, Fig. 2A, left side), indicating rapid convergence. The accuracy surpassed the 95% mark during the first epoch (Fig. 2A, right side), evidencing the model's effectiveness in sequence generation, classification, and clustering (Fig. 2B, C). Nevertheless, 4 epochs should be used for this analysis. The model failed to converge properly whenever the number of epochs was reduced (< 4 epochs), yielding high loss and low accuracy scores. On the other hand, increasing epochs (> 4) did not improve results.

After the resulting graphs were revised, the sequences obtained were further validated by doing a blast against the public databases (GenBank and MalAvi) and the mt genome sequence database published in this investigation (https://github.com/EscalanteLab/HmtG-PacBio-Pipeline.git). Importantly, the coding regions were translated into protein to verify the reading frame and the quality of the sequences using the annotated mt of *P. falciparum* (M76611) as a reference [63].

As an example of the quality of the information obtained, sample 15 was found positive only for *Plasmodium* sp. (lineage BT7, 100% identical) when a traditional *cytb* gene protocol was used [13]. Its electropherograms did not show evidence of mixed or co-infection. However, the results obtained here indicated a mixed infection with two different species of *Leucocytozoon* in addition to the coinfection with *Plasmodium* sp. (Fig. 2C). Specifically, the results from the HmtG-PacBio Pipeline showed four different clusters of sequences (Fig. 2C): one cluster contained and confirmed the *Plasmodium* sp. lineage BT7 sequence (cluster 4) found by using the *cytb* gene protocol, two clusters contained the *Leucocytozoon* spp. lineages BUTJAM19 (cluster 3) and BUTJAM20 (cluster 2) sequences, and a cluster with *Leucocytozoon* sp. sequences (cluster 1) with poor quality (e.g., multiple gaps in coding regions, PCR/sequencing errors). Interestingly, the lineage BUTJAM19, considered new because it has not been reported in any of the databases, was also found (100% identical) in three individuals of Red-tailed Hawks included in this investigation (samples 13–15). The genetic distance between BUTJAM19 (cluster 3) and BUTJAM20 (cluster 2) sequences was 0.14 ± 0.003, suggesting potentially different *Leucocytozoon* species.

**Fig. 3:**
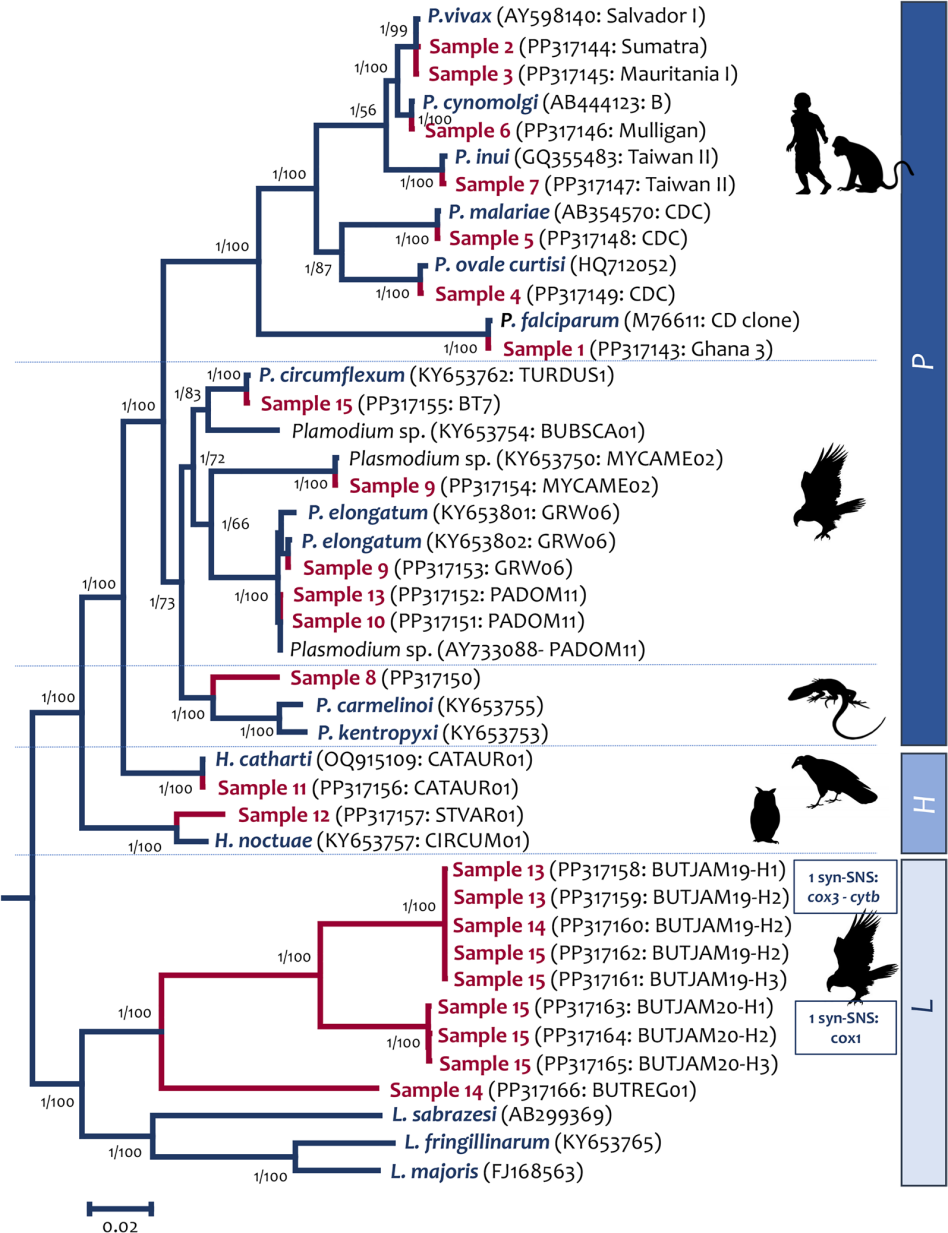
Phylogenetic hypothesis of haemosporidian parasites infecting the selected samples used to test the new mt genome PacBio HiFi sequencing protocol. The phylogenetic tree was computed based on 43 partial parasite mt genomes (5388 bp excluding gaps) belonging to three genera using Bayesian and Maximum Likelihood methods. The values above branches are posterior probabilities/bootstraps respectively. GenBank accession numbers and strains/lineages are provided in parentheses for the sequences used in the analyses. L: *Leucocytozoon*, H: *Haemoproteus*, and P: *Plasmodium*

After a comprehensive analysis of all 15 samples included in this study (Additional file 1: Figure S1), all mt sequences obtained here were compared with what was available in the GenBank. To facilitate and visualize the results, a phylogenetic tree was estimated by two different methods, as was explained in the method section (Fig. 3). Sequences obtained for samples 1 to 7 from humans and macaques were 100% identical to their respective parasite sequences with available data (Fig. 3). Sequence from sample 8 from a Ground Agama (*Agama* *aculeata,* Order Squamata) corresponded to a new potential *Plasmodium* species that can be circulating in Angola–Africa, where this individual was collected in 2017.

Raptor samples 9, and 13 to 15 had mixed and/or co-infections and samples 10 to 12 had single infections (Table 3, Fig. 3). In the case of raptor samples with single infections, *cytb* sequences obtained by the PacBio HiFi protocol matched 100% with the sequences previously obtained using the *cytb* gene protocol [13]. However, in the case of sample 9 (an Osprey), it was previously found positive only for *Plasmodium* sp. lineage MYCAME02 (100% identical) when the *cytb* gene protocol was used. Still, a mixed infection of *Plasmodium* sp. lineage MYCAME02 and *Plasmodium elongatum* lineages GRW06 was detected by the PacBio HiFi protocol (Table 3, Fig. 3). Previously, when the electropherograms for *cytb* gene sequences obtained for samples 13 and 15 were carefully inspected, evidence of mixed/co-infections were found but was not possible to identify the species. The PacBio HiFi protocol, however, detected *Plasmodium* sp. PADOM11 and *Leucocytozoon* sp. lineage BUTJAM19 in sample 13, and *Leucocytozoon* sp. lineages BUTJAM19 and BUTREG01 (one previously reported in MalAvi database) in sample 15 (Fig. 3). Interestingly, this new protocol was able to identify different mt haplotypes for the lineage BUTJAM19, which are differentiated by only one synonymous substitution in *cox3* and *cytb* (outside of the 480 bp *cytb* barcode [11]) genes, and for the lineage BUTJAM20, which are differentiated by only one synonymous substitution in the *cox1* gene. Only a single haplotype was identified for BUTREG01 (Fig. 3). Regardless of this method's accuracy, a cluster of sequences with PCR or putative sequencing errors can be found (e.g., no in-reading frame or a unique haplotype). Thus, carefully inspecting the data (sequences from each cluster obtained) is always advisable, and as the golden rule, new lineages or haplotypes should be confirmed. Actual haplotypes usually appear in more than one individual from a given population, as evidenced by the new lineage BUTJAM19 found in three Red-tailed hawk individuals.

Overall, the average genetic p-distance found, at least in this group of 15 samples, within a cluster was 0.0015 (0.15%), and the average genetic p-distance between clusters was 0.11 (11%).

### In silico experiments

**Fig. 4:**
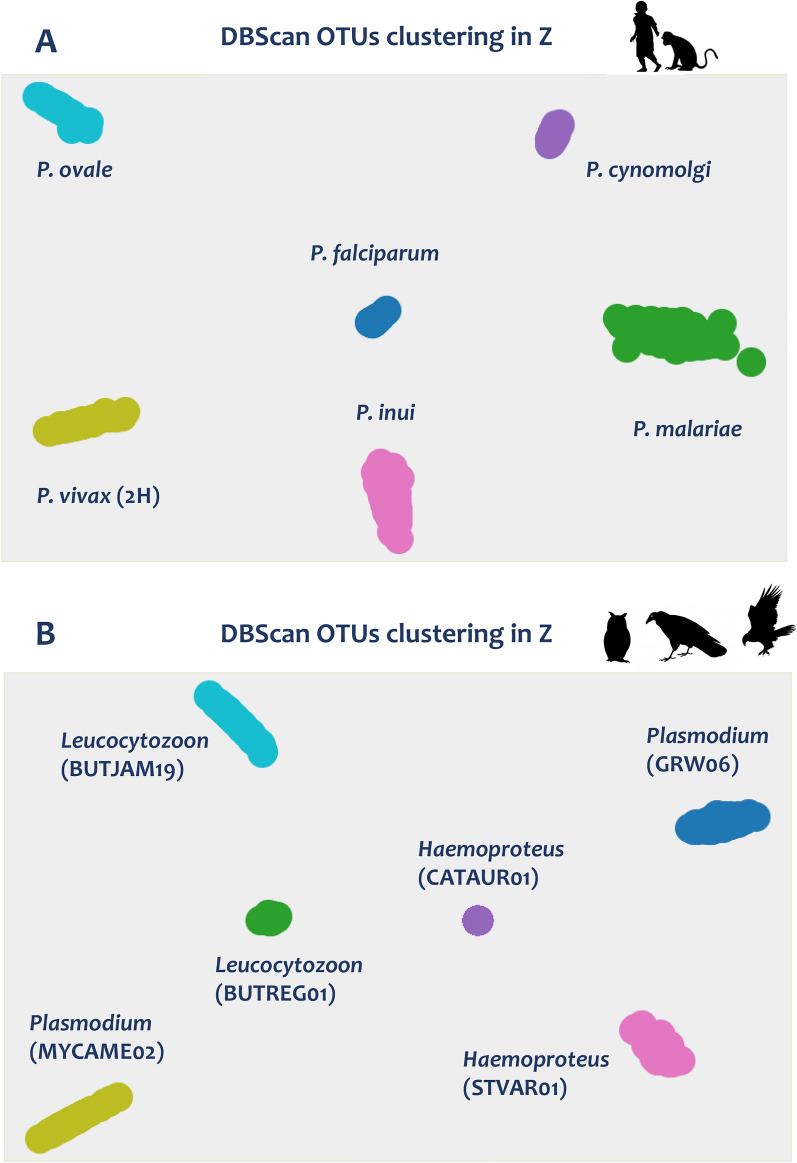
DBScan OTUs clustering in *Z* graph output for the *in-silico* experiments using **A** primate malaria parasites and** B** avian haemosporidian parasites. Species and/or lineage names are indicated for each cluster. Each color corresponded to a different species

As expected, six clusters were recovered from the first experiment that combined all the primate parasites, and each one corresponded to one of the six well-known *Plasmodium* species infecting primates (Fig. 4A). The segregation of the read obtained for each cluster matched 100% with the distribution obtained initially by running each sample independently. Each cluster corresponded to a single *Plasmodium* infection except the *P. vivax*-cluster, which rescued the two *P. vivax* haplotypes combined in this experiment. Six clusters were also recovered in the second in silico experiment performed with raptor samples. Each one corresponded to the lineages found in the four raptor individuals that were combined (Fig. 4B). In this experiment, the lineages found in the two raptor samples with coinfection (samples 9 and 14) were recovered (GRW06/MYCAME02 and BUTREG01/BUTJAM19) and separated into different clusters as expected. All sequences obtained for each cluster also matched 100% with the originally obtained sequences by running each sample independently.

Processing thousands of reads using machine learning algorithms is challenging due to computational constraints. Different dataset sizes using random subsampling without replacement from the original data were tested in a Mac Studio (macOS version 14.1.1) equipped with an Apple M2 Ultra chip with 24 cores (16 performance and 8 efficiency cores) and 192 GB of LPDDR5 RAM. It was found that for samples with a large number of reads (> 5000), a random subsampling of 4000 to 5000 reads (script included in the pipeline) provided results comparable to the original dataset in complex infections. The user may modify the subsample size considering the lower haplotype frequency they want to detect. However, analyzing a small number of reads defeats the purpose of using deep sequencing.

### Sequencing/PCR errors

PacBio HiFi per-read error was consistent with those previously reported for PacBio. According to the Phred read quality scale distribution or Q-value (Additional file 2: Fig. S2A), all sequencing reads had a Q-value greater than 30. This indicates that the maximum expected error rate is one per 1000 nucleotides, equivalent to 0.1%. Second, the observed average error rate was 0.2% per read (with a minimum of 0.03% and a maximum of 0.46%). Thus, a 30X coverage seems adequate to obtain reliable results. Although the error rate per nucleotide site was low, an accumulation of errors at sites with repetitive adenine (A) or thymine (T) nucleotides was detected in only one sample (sample 15). In addition, the read lengths correspond to the expected size of the amplicon, a nearly complete mitochondrial genome (≈ 6 kb) (Additional file 2: Fig. S2B). Lastly, the GC content of the reads, which exhibited a distribution between 30 to 34% (Additional file 2: Fig. S2C), provided further evidence of no contamination from the host (vertebrates GC content distribution of 36 to 50%, Genome List–Genome–NCBI (nih.gov)).

## Discussion

The method described here allows for studying the haemosporidian mt genome with high accuracy. Since the length of this genome can be covered with just one read, it reduces the risk of assembling chimeras or “consensus” sequences for samples that may have mixed and/or coinfections that cannot be detected by the most common standards methods (microscopy and/or direct Sanger sequencing) [2, 3, 10, 13, 15]. As a result, this protocol detects mixed and/or co-infections that may have different levels of parasitaemia (Fig. 2) as well as different lineages and haplotypes that could be present in low frequency, as was evidenced by the results obtained for sample 9 and/or 15, respectively (Fig. 3).

The primers designed here allow for the amplification of a wide variety of haemosporidian species belonging to the most common genera (*Plasmodium*, *Haemoproteus*, and *Leucocytozoon*) that can be found in different vertebrate host species (Tables 1 and 3). Although more data is needed, the results also suggest no evidence of specific primer binding affinity for a particular haemosporidian genus, given that it can detect a co-infection of *Leucocytozoon* and *Plasmodium* in the same sample. It is worth noting that this set of mt primers, in addition to a new *Plasmodium* sp. from an Angolan lizard, allowed the sequence of the mt genome of recently discovered divergent lineages, such as *Haemoproteus catharti* (Fig. 3) and *Haemoproteus pulcher*, whose taxonomy is still unclear [37]. Thus, these primers should perform very well in most haemosporidian species.

Patterns of the complexity of infection are well known for human malaria parasites e.g., [64, 65]; however, there is little known about it in other haemosporidians infecting wildlife, including non-human primates that may harbour zoonotic malarias [25, 26]. So far, using standard cloning methods, some mixed infections have been detected and separated, but it is a laborious and expensive approach if multiple samples are studied [12]. Thus, the method proposed is an accurate alternative to characterize the haemosporidian species assemblages associated with different vertebrate host populations.

Diagnostics involves the detection of defined species, as is typical in human malaria epidemiology. Considering that premise, the proposed methodology is not a diagnostic method, but rather an approach for studying haemosporidian species diversity and discovering putative new species or genotypes by sequencing using the mitochondrial genomes. This method suits various research agendas, from Haemosporida biodiversity assessments to field studies documenting zoonotic malaria from large sets of positive samples. It also allows the use of the mitochondrial genome for studying population dynamics when using a single no recombinant locus is appropriate [28, 29, 66]

It is worth noticing that the local blast search seeks to provide the researcher with a tool to explore and understand the data. However, it is not intended to be an automatic criterion for species identification or delimitation. As the data become richer and more sequences are linked to described species, it may be possible to incorporate a species delimitation algorithm. The investigator can change the reference file containing the mt genome, offering the flexibility of including unpublished data.

Despite its initial high cost, the primers designed here allow the amplification and multiplexing of up to 192 different host samples in one SMRT cell, significantly reducing the cost per sample as part of large surveys. Still, it will be more expensive and laborious than cloning in studies that require a few samples. Also, researchers may be interested in putative single infections or common haplotypes because they can only observe one morphospecies, disregarding variants that may be in low frequency. This method is more laborious than traditional *cytb* gene detection protocols as the PCR steps require multiple primer sets rather than a unique pair and a library prep step. Also, there is a learning curve when using the pipeline. However, in the appropriate context, the cost is considerably low per sequence, given the amount and quality of the data yield.

In conclusion, this robust, high-throughput method can accurately characterize haemosporidian species assemblages and perform genotyping by sequencing targeting their mitochondrial genome. As such, this method allows for studying multiple infections and co-infections, data that is seldom available from non-human hosts. Although a single locus approach, the data quality provides a robust assessment of a species pool that can be used to study parasite biodiversity, biogeography, phylogenetics, and demographic processes, including population structure.

### Supplementary Information

**Additional file 1: Figure S1.** HmtG-PacBio Pipeline graph output for each sample used in this study. (A) Visualization of the training process, (B) mean (μ: black unfilled dots), and standard deviation (σ: blue filled dots) in *Z*, and (C) DBScan OTUs clustering in *Z***. **Species/lineages names are indicated for each sample. See Table 3 for details.

**Additional file 2**: **Figure S2.** Quality (A), read length (B), and GC content distributions (C) for all sequenced samples included in this study.

## Data Availability

All sequences generated and/or analyzed during the current study are available in the GenBank database under the following accession numbers: PP317143—PP317166. The pipeline and database are available here: https://github.com/EscalanteLab/HmtG-PacBio-Pipeline.git.
