# Supplementary material for: A new long-read mitochondrial-genome protocol (PacBio HiFi) for haemosporidian parasites: a tool for population and biodiversity studies
Source: Malar J. 2024 May 4;23:134. doi: 10.1186/s12936-024-04961-8 (PMC11069185; doi:10.1186/s12936-024-04961-8)

Additional information

A new long-read mitochondrial-genome protocol (PacBio HiFi) for haemosporidian parasites: A tool for population and biodiversity studies.

M. Andreína Pacheco[1,*,&], Axl S. Cepeda[1,&], Erica A. Miller[2], Scott Beckerman[3], Mitchell Oswald[3], Evan London[4], Nohra E. Mateus-Pinilla[4,5,6,7], and Ananias A. Escalante[1,*].

[1]Biology Department/Institute of Genomics and Evolutionary Medicine (iGEM), Temple University, Philadelphia, Pennsylvania 19122-1801, USA.
[2]Wildlife Futures Program, University of Pennsylvania, Kennett Square, PA 19348.
[3]USDA Wildlife Services, Springfield, IL. 62711, USA
[4]Department of Animal Sciences, University of Illinois at Urbana-Champaign, Urbana, IL 61801, USA.
[5]Illinois Natural History Survey-Prairie Research Institute, University of Illinois at Urbana-Champaign, Champaign, IL 61820, USA.
[6]Department of Natural Resources and Environmental Sciences, University of Illinois at Urbana-Champaign, Champaign, IL 61820, USA.
[7]Department of Pathobiology, College of Veterinary Medicine, University of Illinois at Urbana-Champaign, Urbana, IL 61802, USA.

*Corresponding authors:
M. Andreína Pacheco: Maria.Pacheco@temple.edu
Biology Department/Institute of Genomics and Evolutionary Medicine (iGEM), Temple University (SERC - 645), 1925 N. 12th St. Philadelphia, PA 19122-1801, USA.

Ananias A. Escalante: Ananias.Escalante@temple.edu
Biology Department/Institute of Genomics and Evolutionary Medicine (iGEM), Temple University (SERC - 653), 1925 N. 12th St. Philadelphia, PA 19122-1801, USA.

&Equal contribution

Additional file 1: Figure S1. HmtG-PacBio Pipeline graph output for each sample used in this study. (A) Visualization of the training process, (B) mean (μ: black unfilled dots) and standard deviation (σ: blue filled dots) in *Z*, and (C) DBScan OTUs clustering in *Z***.** Species/lineages names are indicated for each sample. Black solid dots are noise. Different clusters in a sample are shown in different colors. See Table 3 for details.

1. *Plasmodium falciparum* (Ghana 3)

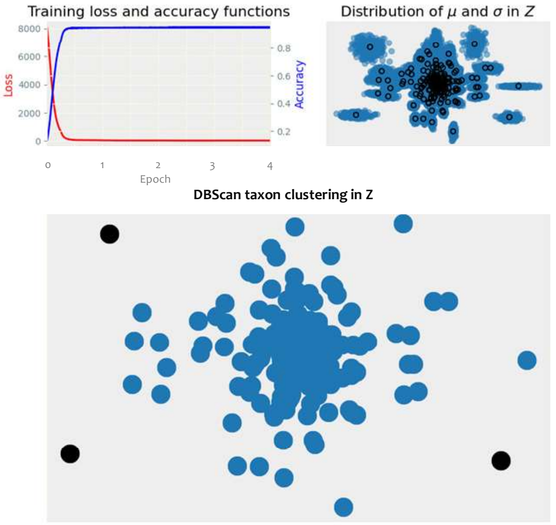

2. *Plasmodium vivax* (Sumatra)

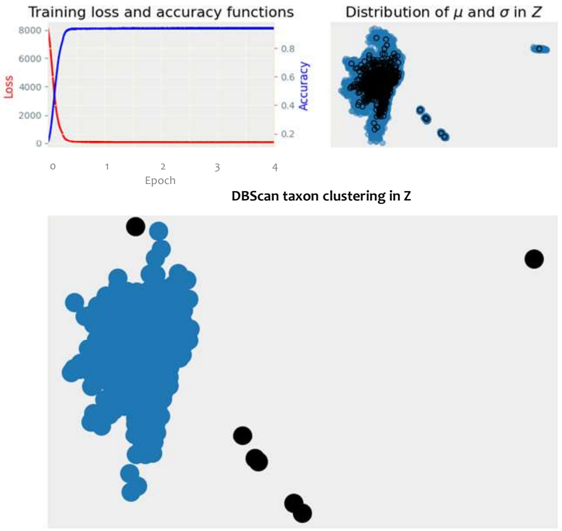

3. *Plasmodium vivax* (Mauritania)

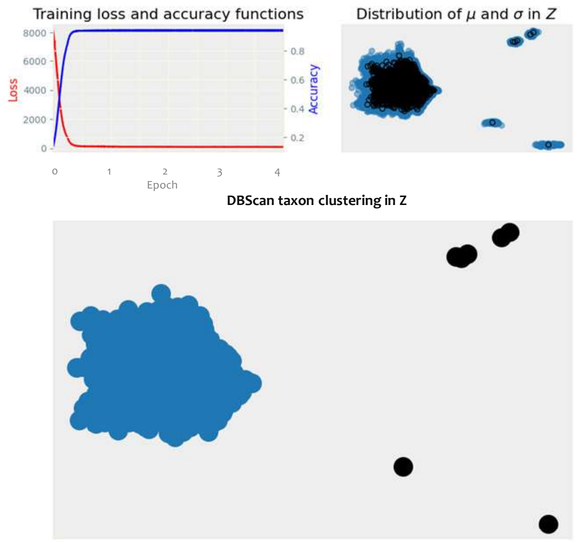

4. *Plasmodium ovale*

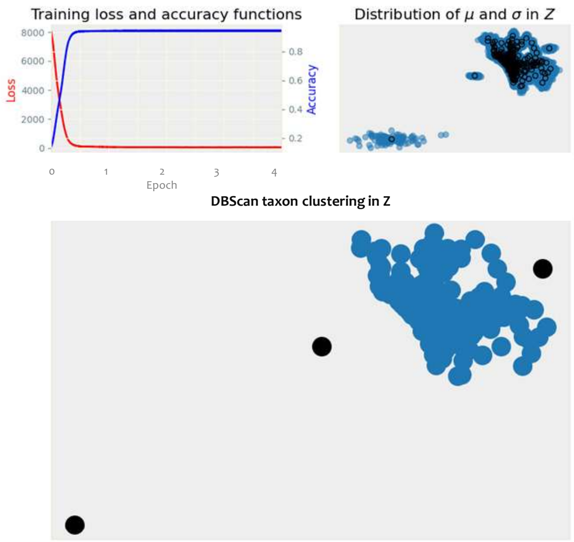

5. *Plasmodium malariae*

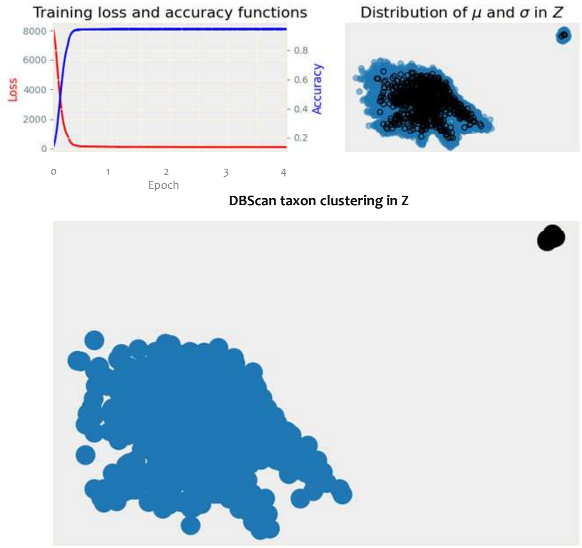

6. *Plasmodium cynomolgi* (macaque)

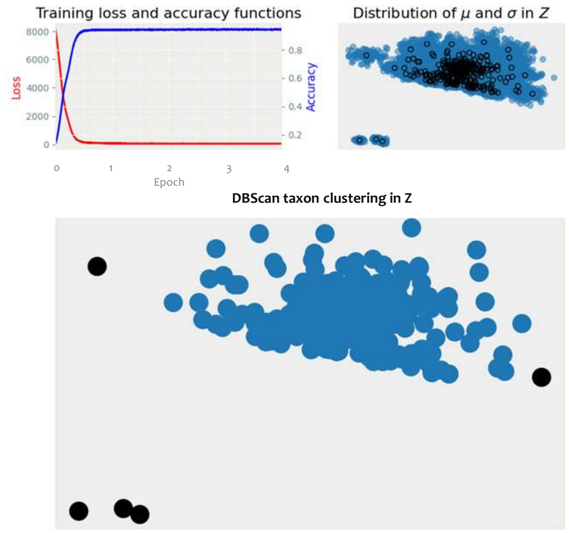

7. *Plasmodium inui* (macaque)

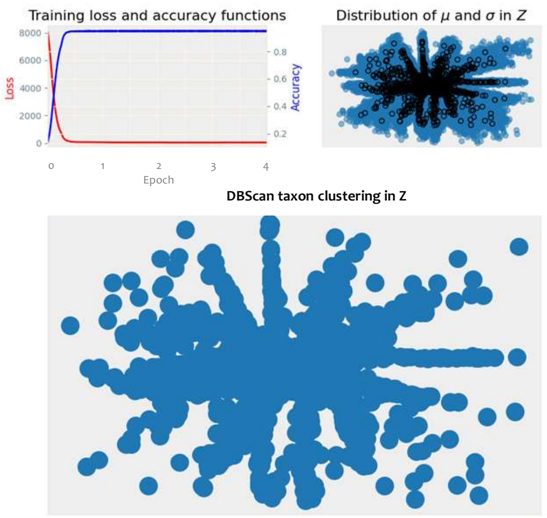

8. *Plasmodium sp.* (*Agama aculeata*)

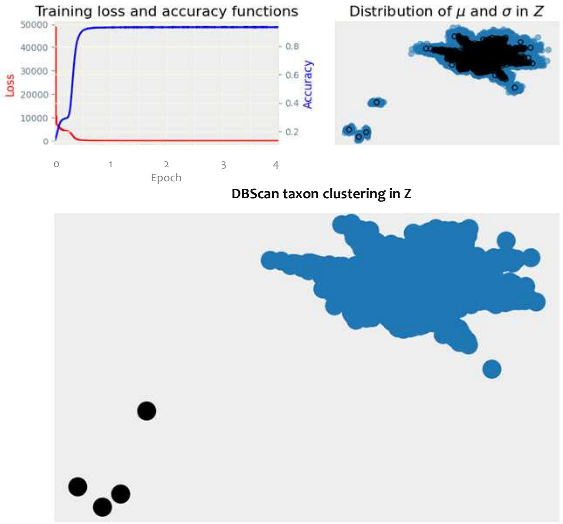

9. *Plasmodium sp.* (Osprey)

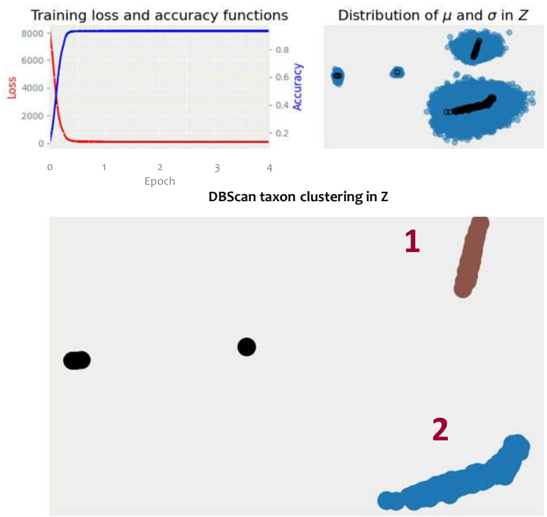

10. *Plasmodium sp.* (Eastern Screech Owl)

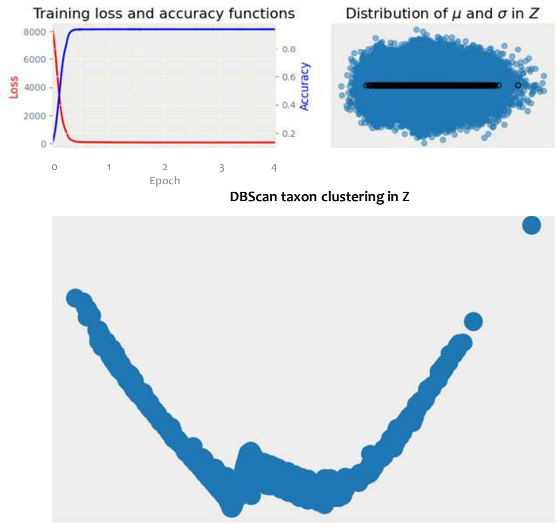

11. *Hemoproteus catharti* (Turkey Vulture)

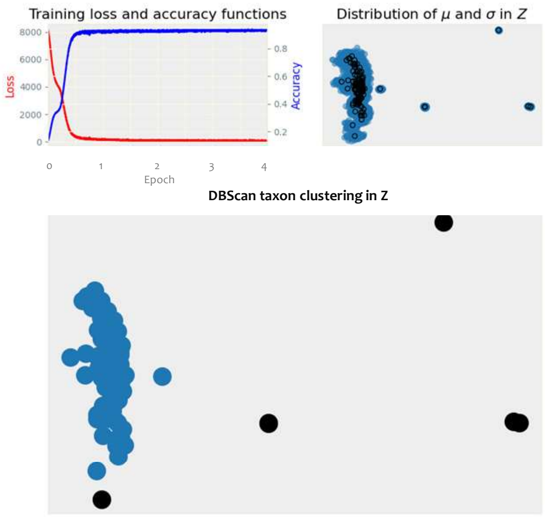

12. *Hemoproteus* sp. (Great horned Owl)

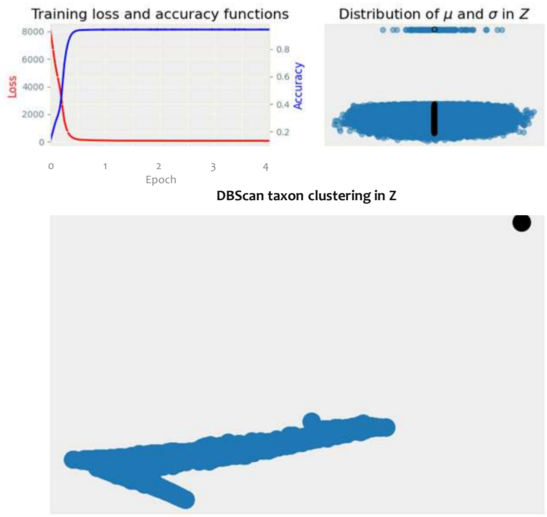

13. *Plasmodium elongatum* (Red-tailed Hawk)

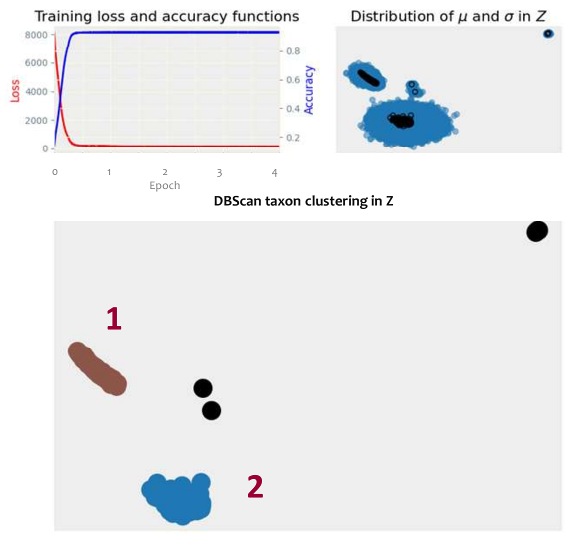

14. *Leucocytozoon* sp. (Red-tailed Hawk)

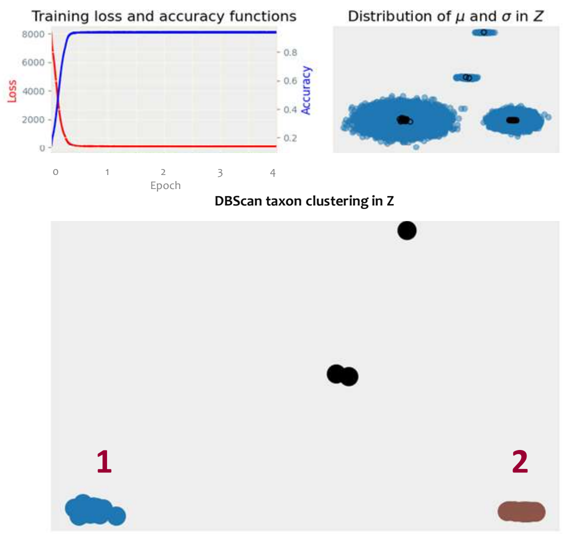

Supplement: Supplementary file 1 — Additional file 1: Figure S1. HmtG-PacBio Pipeline graph output for each sample used in this study. (A) Visualization of the training process, (B) mean (μ: black unfilled dots), and standard deviation (σ: blue filled dots) in Z, and (C) DBScan OTUs clustering in Z. Species/lineages names are indicated for each sample. See Table 3 for details. [file 12936_2024_4961_MOESM1_ESM.pdf]
